# An Infrared and Visible Image Alignment Method Based on Gradient Distribution Properties and Scale-Invariant Features in Electric Power Scenes

**DOI:** 10.3390/jimaging11010023

**Published:** 2025-01-13

**Authors:** Lin Zhu, Yuxing Mao, Chunxu Chen, Lanjia Ning

**Affiliations:** State Key Laboratory of Power Transmission Equipment Technology, School of Electrical Engineering, Chongqing University, Chongqing 400044, China; zhulin030617@163.com (L.Z.); chunxu2chen@stu.cqu.edu.cn (C.C.); ninglanjia2323@gmail.com (L.N.)

**Keywords:** image alignment, infrared and visible image, electricity inspection, gradient direction characterisation

## Abstract

In grid intelligent inspection systems, automatic registration of infrared and visible light images in power scenes is a crucial research technology. Since there are obvious differences in key attributes between visible and infrared images, direct alignment is often difficult to achieve the expected results. To overcome the high difficulty of aligning infrared and visible light images, an image alignment method is proposed in this paper. First, we use the Sobel operator to extract the edge information of the image pair. Second, the feature points in the edges are recognised by a curvature scale space (CSS) corner detector. Third, the Histogram of Orientation Gradients (HOG) is extracted as the gradient distribution characteristics of the feature points, which are normalised with the Scale Invariant Feature Transform (SIFT) algorithm to form feature descriptors. Finally, initial matching and accurate matching are achieved by the improved fast approximate nearest-neighbour matching method and adaptive thresholding, respectively. Experiments show that this method can robustly match the feature points of image pairs under rotation, scale, and viewpoint differences, and achieves excellent matching results.

## 1. Introduction

Power inspection is a key method for ensuring the safe and stable operation of transmission lines and power equipment, in which automated inspection technologies such as UAV aerial photography are gradually replacing the traditional manual inspection methods and are widely used in the intelligent fault diagnosis system of power equipment [1]. With the rapid development of automation technology and artificial intelligence, guiding machines to parse perceptual data, identify differences or associations between multiple visual targets, and make optimal decisions under specific conditions has become the core topic of computer vision research. Image alignment, also known as image matching, is a fundamental task in computer vision applications that aims to identify and correspond to the same or similar structures and contents from two or more images [2].

**Figure 1:**
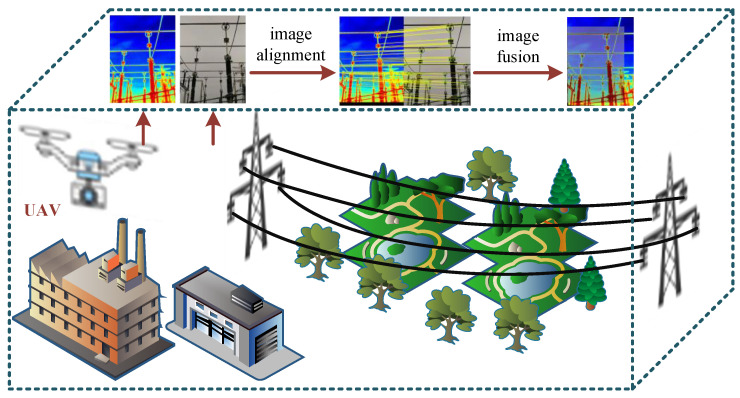
The drone captures infrared and visible light images of the same power scene and performs image alignment for automated detection.

The core goal of image alignment is to construct spatial mapping relationships between multiple images of a similar scene captured by different sensors and from various viewpoints to achieve the same object’s alignment in heterogeneous source images [3]. As shown in Figure 1, a UAV can capture multi-source image information such as infrared and visible light images in the power scene to obtain a more comprehensive description of the scene. Infrared images reveal the thermal state of the equipment, helping to detect abnormalities such as overheating and faults, while visible light images provide intuitive visual information such as the form, markings, and precise location of the equipment. Through the automated alignment of these two images, early warning and prevention of power equipment faults can be achieved, effectively reducing the negative impact of faults on grid operation and economic losses. Integrating the automatic alignment technology of infrared and visible light images into the grid intelligent inspection system can not only improve inspection efficiency and reduce manpower costs but also reduce the direct physical contact between operation and maintenance personnel and power equipment, thus reducing the safety risks associated with the high-voltage electric environment and overhead work.

Heterogenic image alignment is a challenging research task in the field of computer vision. Due to a variety of factors such as shooting conditions, shooting angle, sensor resolution, etc., there are often significant brightness and contrast differences, geometric deformation, scale inconsistency, and noise interference between image pairs, which makes it difficult to achieve ideal results in direct alignment. Infrared images are based on thermal radiation imaging of the target, which can effectively detect and identify scene targets, but the resolution is usually low and lacks detailed texture information. On the contrary, visible light images conform to human visual habits, with high spatial resolution and clear texture details; however, the imaging is susceptible to ambient lighting conditions [4]. A single infrared or visible light image is difficult to meet the needs of practical applications, and it is necessary to integrate infrared and visible light images to achieve complementary information. Therefore, the development of efficient, high-precision image alignment technology is an important prerequisite for the deep fusion of infrared and visible image information.

For specific electric power inspection application scenarios, infrared images show the characteristics of temperature concentration, similar contour features, low contrast, etc., coupled with the fact that the multi-source image pairs acquired by the inspection equipment are prone to rotations and scale variations, which further increases the difficulty of aligning infrared and visible images in electric power scenarios. The SIFT algorithm, as a classic algorithm in the field of image alignment, has scale invariance under image translation, rotation, scaling, and other transformations and can better maintain image features. Therefore, we have studied the infrared and visible light image alignment methods for electric power scenarios to achieve good results.

In this paper, we take the point feature matching method as the research object and propose a normalised directional Histogram of Orientation Gradients [5] combined with scale-invariant feature transform (HOFT) for infrared and visible image registration algorithms. First, the edge information of infrared and visible source images is extracted by the Sobel edge detection operator [6] to improve the similarity of the image pairs. Second, scale-invariant feature points are extracted using a contour curvature-based CSS corner point detector [7]. Then, the gradient information distribution property with directional gradient histogram is introduced within the SIFT framework to establish an improved scale-invariant feature transform descriptor. Finally, the bilateral fast approximate nearest neighbour (FLANN) [8] search matching method with intrinsic information error compensation is utilised for preliminary matching and adaptive thresholding, and the RANSAC algorithm is used for precise matching to obtain the final correct matching point pairs and the parameters of the similar transform model.

The main contributions of this paper are threefold:

1. A normalised directional histogram of orientation gradients combined with scale-invariant feature transform (HOFT) is proposed as an alignment algorithm for infrared and visible images;
2. We propose a feature descriptor that can overcome the effects of scale, rotation, and viewpoint differences to accurately describe key information in visible and infrared images;
3. We optimise and design an efficient matching strategy to obtain denser matching pair information and more accurate image alignment results.

The rest of the paper is organised as follows: We describe the relevant background knowledge in Section 2 and the details of our matching algorithm in Section 3. In Section 4, we conduct experiments on publicly available datasets of visible and infrared images of power equipment and discuss the results. The paper is summarised in Section 5.

## 2. Related Work

Existing image alignment algorithms are mainly divided into two types: region-based and feature-based.

### 2.1. Region-Based Image Alignment Method

Image region-based research methods rely on an accurate and appropriate similarity metric mechanism. This process involves an in-depth analysis of the grey values in the image, aiming to quantify the degree of similarity between the target image and the reference image and to determine the optimal matching position between the pairs of images to construct a pixel-level accurate correspondence [2]. To achieve this goal, researchers usually presuppose a transformation model and choose advanced statistics such as Mutual Information (MI) or Normalised Cross-Correlation (NCC) as similarity metrics. These metrics can effectively estimate and optimise the parameters of the transformation model to achieve accurate alignment of the two images in the common region [9].

From a methodological point of view, region-based image alignment algorithms show the advantage of being intuitive and easy to understand. However, its application faces several challenges. First and foremost, the algorithm is highly dependent on the complete pixel information of the image, which means that any form of noise or geometric distortion in the image may significantly affect the accuracy of the alignment. Furthermore, due to the need to process and analyze detailed information of all pixels in an image, such algorithms are often accompanied by high computational complexity, which largely limits their breadth and efficiency in practical applications [10]. Therefore, despite the attractiveness of region-based image alignment algorithms at the theoretical level, they are used relatively infrequently in current practical applications, especially in scenarios with stringent requirements on computational resources and time costs.

### 2.2. Feature-Based Image Alignment Method

Feature-based image alignment algorithms discard the comprehensive reliance on global information about an image pair and instead focus on highly salient and representative local features in the image. The transformation model between image pairs is computed by matching between local features, commonly used image features include point, line, and surface features [2]. For example, feature elements such as corner points, edge points, and centre points of closed regions in an image are extracted as key points. The image features are then described by choosing suitable descriptors, establishing a strategy to achieve keypoint matching, and finally solving the alignment parameters.

The scale-invariant feature transform algorithm proposed by David Lowe [11] establishes the basic framework of point-based feature alignment methods, which is the most classical point-matching method and has received numerous extended applications. Jiang [12] proposed the CAO-C2F method, which calculates the main direction of the contour and combines it with the improved scale-invariant feature transform descriptor for bilateral matching to realise the infrared and visible power image alignment. Speeded Up Robust Features (SURF) [13] was proposed based on SIFT; this was achieved by relying on integral images for image convolution and using a Hessian matrix-based measure for the detector and a distribution-based descriptor. Next, an alignment algorithm called Edge-Guided Speeded-Up Robust Features [14] combines local and global information into a single descriptor. The regional information is extracted using SURF, while the worldwide information is represented by the shape context of the edges, gathering more public information during the alignment process. Chen [15] proposed a method for the computation of the squared gradient principal directions and a local intensity invariant feature descriptor (PIIFD), which extends the scale-invariant feature transformation method to image matching in multimodal retinas. However, due to significant differences in resolution and appearance, it is usually unable to correctly align infrared and visible image pairs, so a scale-invariant PIIFD [16] feature computed for corner points was proposed. Corner points are first extracted as candidate control points since they are usually sufficient and uniformly distributed throughout the image domain. Then, PIIFD is computed for all corner points and matched based on descriptor similarity and locally preserved geometric constraints. However, this method has some limitations and maintains the matching effect only for local features. Lu [17] proposed an L-FAST algorithm. Aiming to solve the difficult problem of extracting edges from the dark areas of uneven brightness images, the effect of uneven brightness is effectively reduced by improving the traditional Canny algorithm, including gradient computation. Andriyanov [18] combined multi-temporal and multi-angle images of architectural structures based on a high-speed pseudo-gradient algorithm to identify key changing feature points. Harris [19] proposed an artificial definition of corner points, which has poor scale-invariant performance despite its better image rotation invariance. A novel deep learning network is utilised to generate high-quality pseudo-optical images from images [20], and feature points are detected and extracted for matching using the multi-scale Harris algorithm. There are also some methods focusing on the aspect of contour features; for example, Si [21] proposed an alignment algorithm for thermal infrared and visible images of apples based on the alignment of feature points with an active contour model, where the set of feature points is constructed by active contour segmentation curves and linear interpolation methods. This leads to improved alignment results for images with more obvious contours. In addition, since most of the scenes are not planar in practical applications, nonlinear transformations are more suitable than affine transformations. Min [22] proposed an Enhanced Affine Transform (EAT) for non-rigid infrared and visible image alignment, which describes the regular pattern of the body image to non-rigid and global deformations based on local features for non-rigid image alignment through a combination of affine and polynomial transforms.

Currently, feature-based image alignment algorithms significantly reduce the amount of image data that are processed while effectively retaining key feature information in the image. However, when processing infrared images and visible light images, they show significant differences in imaging modality and greyscale distribution, which leads to a significant weakening of the inter-pixel correlation [23]. In addition, the images may be affected by multiple factors such as the shooting scene, resolution differences, and changes in viewing angle, which together result in significant differences in spectral characteristics and varying degrees of misalignment between the images.

## 3. Methods

**Figure 2:**
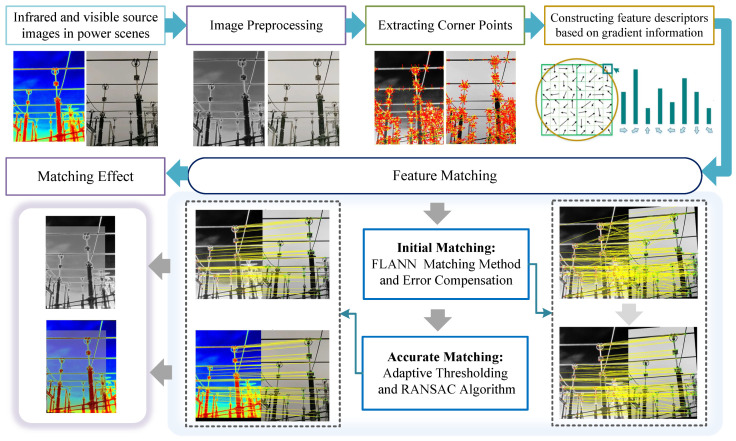
Flowchart of the proposed HOFT image alignment method.

In this section, we describe the proposed image alignment algorithm in detail. First, the edge contours of the image are detected using the Sobel detector, and then the feature points are extracted using the CSS corner point detection algorithm. Next, a scale-invariant feature transform descriptor based on HOG feature points is constructed. Finally, preliminary matching is carried out by the FLANN algorithm and error compensation of inherent information of feature points, and then the adaptive threshold is set and the RANSAC algorithm is used to realise accurate matching. The overall matching process of our method is shown in Figure 2.

### 3.1. Edge Detection and Feature Extraction

**Figure 3:**
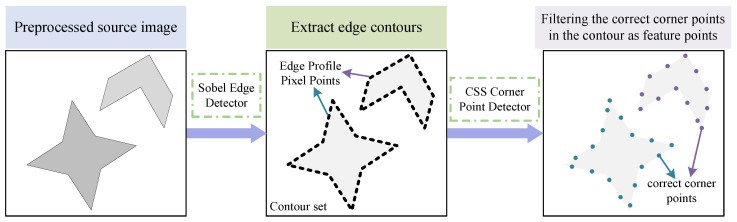
Process of edge detection and extraction of correct corner points.

In the edge detection process, we first identify the pixels in the image whose grey values have changed significantly and aggregate these pixels into a specific set of pixels [24]. Subsequently, we rely on this pixel collection to accurately define and identify the key feature structures in the original image, as shown in Figure 3. Finally, the edge features of the image are obtained by analysing and extracting the information of these feature structures [25]. In this paper, we use the Sobel operator to detect the edge information. The Sobel operator contains two sets of 3 × 3 matrices, horizontal and vertical matrices, which are convolved with the image to obtain the approximation of the luminance difference of horizontal and vertical matrices. We extract the edge information by using the weighted difference of the pixel greyscale in the upper and lower neighbourhoods, left and right neighbourhoods. We randomly select a 3 × 3 region in the original image, and $G_{L}$ and $G_{V}$ denote the grey value of the image after horizontal and vertical edge detection, respectively. The formula is as follows:

(1) $$I=\left(\begin{array}{ccc} f_{\left(x-1,y-1\right)} & f_{\left(x-1,y\right)} & f_{\left(x-1,y+1\right)}\\ f_{\left(x,y-1\right)} & f_{\left(x,y\right)} & f_{\left(x,y+1\right)}\\ f_{\left(x+1,y-1\right)} & f_{\left(x+1,y\right)} & f_{\left(x+1,y+1\right)} \end{array}\right)$$

(2) $$G_{L}=\left(\begin{array}{ccc} -1 & 0 & 1\\ -2 & 0 & 2\\ -1 & 0 & 1 \end{array}\right)*I$$

(3) $$G_{V}=\left(\begin{array}{ccc} 1 & 2 & 1\\ 0 & 0 & 0\\ -1 & -2 & -1 \end{array}\right)*I$$

where $f_{\left(x,y\right)}$ denotes the grey value of point (x,y) in the image. The horizontal and vertical grey values of any pixel of the image are combined by the following formula to calculate the grey size of the point.

(4) $$G\left(x,y\right)=\sqrt{G_{L}^{2}+G_{V}^{2}}\approx \left|G_{L}\right|+\left|G_{V}\right|$$

The pixel gradient can be approximated according to the above equation. By choosing a suitable threshold, the gradient approximation is compared and the points larger than the threshold are the edge points [26]. Define the original contour curve pixel coordinates set in a single image:

(5) $$C={\left\{\Gamma^{j}|\Gamma^{j}=\left\{p_{1}^{j},p_{2}^{j},p_{3}^{j},\cdots ,p_{n}^{j}\right\}\right\}}_{i=1}^{N}$$

where $\Gamma^{j}$ denotes the *i*th contour in *C*, *n* is the number of pixel points in the *i*th contour, and *N* is the number of contours in the image. A plane curve in an image is represented as the trajectory of a point on a plane, defining a plane curve as follows:

(6) $$l\left(\upsilon \right)=\left\{x(\upsilon ),y(\upsilon )\right\}$$

Considering the presence of burrs and the effect of noise, the plane curve *l* is smoothed and filtered with a Gaussian function with scale parameter σ:

(7) $$\begin{array}{cc} l(\upsilon ,\sigma ) & =\left\{X(\upsilon ,\sigma ),Y(\upsilon ,\sigma )\right\} \end{array}$$

(8) $$\begin{array}{cc} X(\upsilon ,\sigma ) & =x(\upsilon )⊗G(\upsilon ,\sigma ) \end{array}$$

(9) $$\begin{array}{cc} Y(\upsilon ,\sigma ) & =y(\upsilon )⊗G(\upsilon ,\sigma ) \end{array}$$

⊗ stands for convolution operation, G(υ,σ) is the Gaussian kernel function. The curvature of a plane curve l(υ,σ) can be defined as follows:

(10) $$K\left(\upsilon ,\sigma \right)=\frac{X^{\prime }\left(\upsilon ,\sigma \right)Y^{\prime \prime }\left(\upsilon ,\sigma \right)-X^{\prime \prime }\left(\upsilon ,\sigma \right)Y^{\prime }\left(\upsilon ,\sigma \right)}{{\left(X^{\prime }{\left(\upsilon ,\sigma \right)}^{2}+Y^{\prime }{\left(\upsilon ,\sigma \right)}^{2}\right)}^{3/2}}$$

where,

(11) $$\begin{array}{cc} X{\left(\upsilon ,\sigma \right)}^{\prime } & =\frac{d(x(\upsilon )⊗G(\upsilon ,\sigma ))}{d\upsilon }=x\left(\upsilon \right)⊗G{\left(\upsilon ,\sigma \right)}^{\prime } \end{array}$$

(12) $$\begin{array}{cc} Y{\left(\upsilon ,\sigma \right)}^{\prime } & =\frac{d(y(\upsilon )⊗G(\upsilon ,\sigma ))}{d\upsilon }=y\left(\upsilon \right)⊗G{\left(\upsilon ,\sigma \right)}^{\prime } \end{array}$$

(13) $$\begin{array}{cc} X{\left(\upsilon ,\sigma \right)}^{\prime \prime } & =\frac{d^{2}\left(x\left(\upsilon \right)⊗G\left(\upsilon ,\sigma \right)\right)}{d^{2}\upsilon }=x\left(\upsilon \right)⊗G{\left(\upsilon ,\sigma \right)}^{\prime \prime } \end{array}$$

(14) $$\begin{array}{cc} Y{\left(\upsilon ,\sigma \right)}^{\prime \prime } & =\frac{d^{2}\left(y\left(\upsilon \right)⊗G\left(\upsilon ,\sigma \right)\right)}{d^{2}\upsilon }=y\left(\upsilon \right)⊗G{\left(\upsilon ,\sigma \right)}^{\prime \prime } \end{array}$$

$G{\left(\upsilon ,\sigma \right)}^{\prime }$ and $G{\left(\upsilon ,\sigma \right)}^{\prime \prime }$ are the first- and second-order derivatives of the Gaussian function G(υ,σ), respectively. First, the curvature scale space corner detection algorithm performs curve smoothing and curvature estimation on the extracted image edges and determines the corner points by selecting the curvature extreme points [27]. Second, a larger scale $\sigma_{h}$ is used to calculate the curvature on the contour curve, and points with absolute curvature values greater than a set threshold ε are used as candidate corner points, and if the curvature value of a candidate corner point is twice the curvature value at the neighbouring local minima, it is taken as the correct corner point.

### 3.2. Generate Feature Descriptors

**Figure 4:**
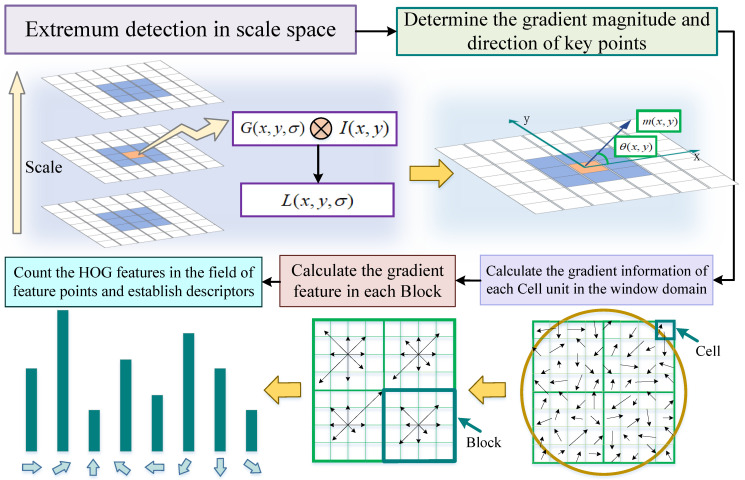
Computation process of feature descriptors.

Generating feature descriptors refers to extracting feature vectors from multiple images to be matched that are independent of rotation, viewing angle, and scale scaling. As shown in Figure 4, in this paper, the Gaussian kernel function is used to perform multi-scale filtering on the image and detect extreme points on multiple scale spaces. The  descriptors of the feature points are determined by the directional gradient histogram [28], which in turn realises the image alignment between infrared and visible light in the electric power scene. The main steps are as follows:

The current detected pixel is compared with the neighbouring 8 pixels on the same scale and the neighbouring 9 × 2 pixels at the corresponding positions on the high and low 2 scales, and if it is the maximum or minimum value, the point is considered to be a feature point of the image at that scale. For the detected feature point, a Gaussian image closest to this scale is calculated based on the feature point scale σ:

(15) $$\begin{array}{cc} L(x,y,\sigma ) & =G(x,y,\sigma )⊗I(x,y) \end{array}$$

(16) $$\begin{array}{cc} G(x,y,\sigma ) & =\frac{1}{2\pi \sigma^{2}}e^{\frac{-(x^{2}+y^{2})}{2\sigma^{2}}} \end{array}$$

L(x,y,σ) is the scale space of the image, which is obtained by convolving a variable scale Gaussian function with the input image, and the orientation parameter is specified for each key point using the gradient direction distribution property of the pixels in the neighbourhood of the key point. The gradient magnitude and magnitude angle of the key points are calculated:

(17) $$\begin{array}{cc} m(x,y) & =\sqrt{{\left(L\left(x+1,y\right)-L\left(x-1,y\right)\right)}^{2}+{\left(L\left(x,y+1\right)-L\left(x,y-1\right)\right)}^{2}} \end{array}$$

(18) $$\begin{array}{cc} \theta (x,y) & =\arctan\frac{L(x,y+1)-L(x,y-1)}{L(x+1,y)-L(x-1,y)} \end{array}$$

The keypoint descriptor is created by calculating the magnitude and direction of the gradient for each image sample point in the region surrounding the keypoint location [29]. The axes are first rotated to the direction of the key point to ensure rotational invariance [30]. Next, the HOG feature point descriptor is constructed.

The basic steps are as follows:

1. An 8 × 8 window neighbourhood is selected centred on the key point, each window represents a pixel in the scale space where the neighbourhood of the key point is located, these are weighted by a Gaussian window, represented by an overlaying circle, the direction of the arrow represents the gradient direction of that pixel, and the length of the arrow represents the gradient magnitude, and the gradient magnitude and direction are calculated for each pixel in the image.
2. Divide the image into Cells and compute the gradient information for each Cell.
3. Equalise the entire rectangular neighbourhood into 4 × 4 sub-neighbourhoods, combine the Cell within each sub-area into a Block, and compute the gradient features within each Block.
4. The neighbourhood window centred on the key point is inwardly sampled, the Block in each sub-neighbourhood is combined to form the HOG features, the feature direction creates the histogram of gradient direction at $45^{\circ }$ intervals.
5. The HOG feature descriptor of the image is obtained by normalisation. The  generated feature vector is first subjected to an initial normalisation process, which usually involves dividing each element in the feature vector by the number of vanes of the feature vector so that its length becomes 1.
  (19) $$A_{norm}=\frac{A}{\sqrt{\sum (A_{i}^{2})}}$$
  *A* is the original feature vector and $A_{norm}$ is the normalised feature vector. To further improve the discriminative nature of the feature descriptors, the normalised feature vector is also truncated. Element values greater than 0.2 in the feature vector are truncated to 0.2 to reduce the impact of too-large feature values on the matching results. After truncation, the length of the eigenvector may change, so it needs to be normalised again by dividing each element in the eigenvector by the number of vanes of the eigenvector so that its length becomes 1.

In particular, the length of each arrow corresponds to the sum of the gradient magnitude near that direction in the region, the direction with the largest cumulative value is the primary direction of the feature point, and the direction with a cumulative value greater than 80 percent of the cumulative value of the primary direction is set as the secondary direction, and a Block of key points may be specified to have 1 primary direction and more than 1 secondary direction. The experiments in this paper use a feature vector of 4×4×8=128 elements for each key point.

### 3.3. Feature Matching

The Fast Library for Approximate Nearest Neighbours(FLANN) search algorithm [31] determines the degree of matching of feature points by calculating the Euclidean distance between the feature point descriptors for the initial matching of feature points. The algorithm generally selects the two points closest to the feature points for calculation. There are *m* feature points in the feature point set *P* of the reference image and *n* feature points in the feature point set $P^{\prime }$ of the image to be matched, denoted as $P=p_{1},p_{2},p_{3},\cdots ,p_{m}$, and $P^{\prime }=p_{1}^{\prime },p_{2}^{\prime },p_{3}^{\prime },\cdots ,p_{n}^{\prime }$, respectively.

First, take a feature point *a* in the feature point set *P* of the reference image and find the two feature points *b* and *c* in the feature point set $P^{\prime }$ of the image to be matched that are closest to the Euclidean distance of the point *a*. Then, the closest Euclidean distance and the second-closest Euclidean distance are obtained as $D_{ab}$ and $D_{ac}$, respectively. The Euclidean distance expression is as follows:

(20) $$D_{MN}=\sqrt{\sum_{i=1}^{64}{\left(\nu_{Mi}-u_{Ni}\right)}^{2}}$$

*M* and *N* are the feature points of the reference image and the matched image point set, respectively, and $D_{MN}$ is the Euclidean distance between point *M* and point *N*. $\nu_{Mi}$ and $u_{Ni}$ denote the values of the *i*th dimension of the feature descriptors of the feature points *M* and *N*.

In this paper, we improve the traditional FLANN algorithm by considering that most correctly matched pairs should have the same rotation angle, scale ratio, and horizontal and vertical displacements in space under a similar transformation model. Therefore, we can use each key point’s inherent position, scale, and orientation information to increase the number of correct matches [32].

The position of key point $p_{i}$ in the reference image is denoted as $\left(x_{i},y_{i}\right)$, the scale is denoted as $Sc_{i}$, and the main direction is denoted as $\theta_{i}$. The position of key point $p_{i}^{\prime }$ in the image to be matched is denoted as $\left(x_{i}^{\prime },y_{i}^{\prime }\right)$, the scale is denoted as $Sc_{i}^{\prime }$, and the main direction is denoted as $\theta_{i}^{\prime }$. The corresponding key point position transformation error $E_{p}\left(i\right)$, scale error $E_{Sc}\left(i\right)$, and relative principal direction error $E_{\theta }\left(i\right)$ in the two images are denoted as follows:

(21) $$\begin{array}{cc} E_{p}\left(i\right) & =∥\left(x_{i},y_{i}\right)-M(\left(x_{i}^{\prime },y_{i}^{\prime }\right),\xi ∥ \end{array}$$

(22) $$\begin{array}{cc} E_{Sc}\left(i\right) & =\left|1-\frac{Sc_{i}^{\prime }}{Sc_{i}}\lambda \right| \end{array}$$

(23) $$\begin{array}{cc} E_{\theta }\left(i\right) & =\left|\Delta \theta_{i}-\Delta \theta_{*}\right| \end{array}$$

(24) $$\begin{array}{cc} \Delta \theta_{i} & =\theta_{i}-\theta_{i}^{\prime } \end{array}$$

*M* is the similar transformation model and ξ is the similar transformation model parameters. λ and $\Delta \theta_{*}$ denote the modal position of the scale ratio and principal direction difference of the reference image and the image to be aligned, respectively. In this paper, we define the error-compensated Euclidean distance $D_{dso}$ considering the location, scale and direction of key points. Therefore, it has better robustness than the general Euclidean distance.

(25) $$D_{dso}\left(i\right)=\left(1+E_{p}\left(i\right)\right)\left(1+E_{Sc}\left(i\right)\right)\left(1+E_{\theta }\left(i\right)\right)\cdot D_{MN}$$

The FLANN algorithm requires a pre-set threshold τ. When the ratio of the error compensation distance between the feature point and the point to be matched and the error compensation distance between the feature point and the next point to be matched is less than the threshold then the matching is successful and greater than the threshold then the matching fails [33]. As shown in the following equation:

(26) $$\frac{D_{dso}\left(a\right)}{D_{dso}\left(b\right)}<\tau$$

**Figure 5:**
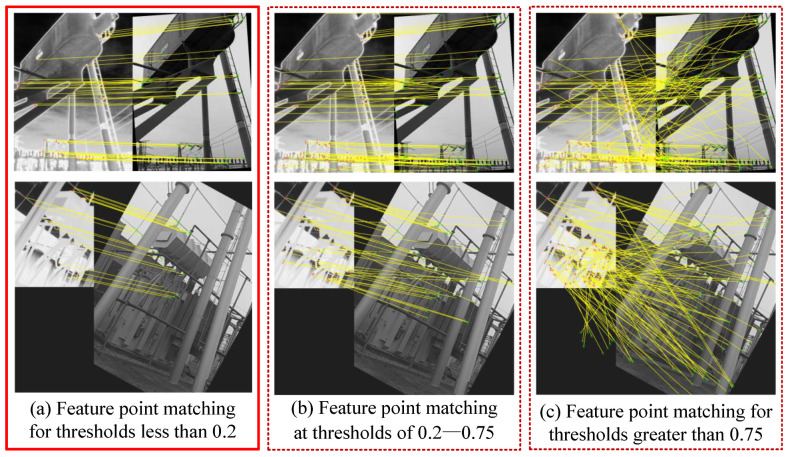
Comparative experimental effect diagram of feature point matching in different threshold ranges. The red solid line box (**a**) indicates that feature point matching achieves good results when the threshold is less than 0.2. The dotted line box (**b**,**c**) indicates the different matching effect graphs when the threshold value is greater than 0.2.

However, the preset thresholds can cause the loss of a large number of feature-matching point pairs to some extent, which affects the matching accuracy. Therefore, this paper proposes a solution to effectively identify and remove false matches by matching accuracy with different thresholds and combining it with the RANSAC algorithm [34]. As shown in the Figure 5.

By analysing the matching results of the image set, we find that when the threshold is set low (less than 0.2), although the matching accuracy is more than 98 percent, it will lead to a significant reduction in the number of retained feature pairs. When the threshold is set too high (greater than 0.75), the false matching rate of the algorithm rises significantly, and the number of false feature pairs increases subsequently. Therefore, in the interval where the threshold value lies in the range of 0.2–0.75, we use the RANSAC algorithm to remove the wrong matching pairs, which not only ensures the correct rate of image alignment but also retains a larger number of feature point pairs. Finally, the set of excellent matching point pairs less than the threshold value of 0.2 is combined with the set of feature point pairs with wrong matching pairs removed to complete the matching.

## 4. Experiments and Discussion

In this section, to validate the effectiveness of our proposed method, we compare it with four other infrared and visible image alignment methods, including PIIFD [16], SURF-RANSAC [13], CAO [12], and HPCO [35]. The methods used for the comparison are implemented based on the MATLAB code and parameters provided by the authors. This experiment is performed on an Intel(R) Core(TM) i5-10400F CPU @ 2.90 GHz hardware platform and MATLAB R2022b software platform.

### 4.1. Datasets and Evaluation Indicators

**Figure 6:**
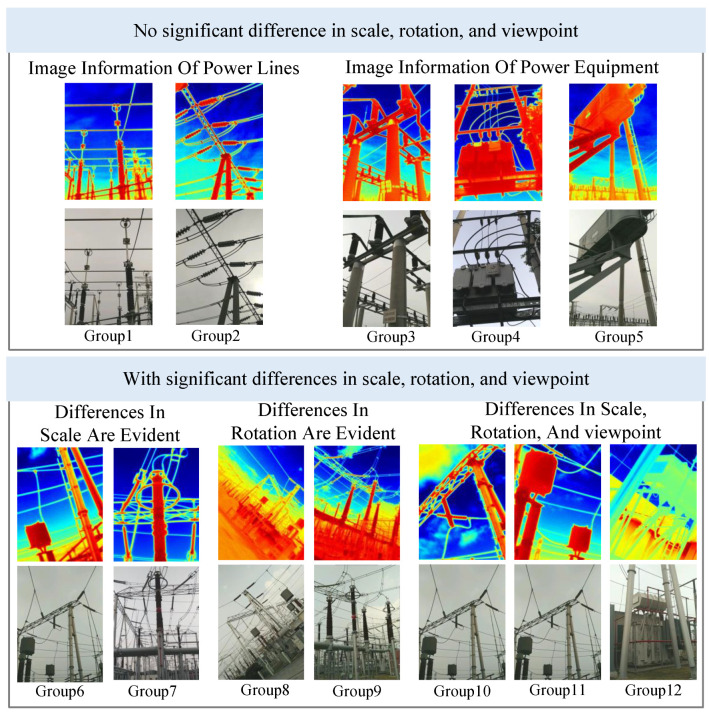
Infrared and visible light source images.

In this paper, we select two groups of visible and infrared images from publicly available datasets [12] with different types of power scenes for the evaluation of image alignment algorithms. Table 1 describes the details of the dataset. The infrared and visible light source images for each group are shown in Figure 6.

Groups 1–5 include five pairs of visible and infrared images without obvious scale, rotation, and viewpoint differences, where image pairs 1 and 2 mainly contain image information about power lines, and image pairs 3, 4, and 5 contain image information about power equipment. Groups 6–12 include seven pairs of visible light and infrared images with obvious scale, rotation, and viewpoint differences. Among them, 6 and 7 are image pairs with obvious scale but insignificant scale and viewpoint differences. Groups 8 and 9 are pairs with obvious rotations differences but insignificant rotations and viewpoint differences. Groups 10, 11, and 12 are pairs with obvious scale, rotation, and viewpoint differences. The resolutions of all visible and infrared image pairs are 800 × 600 and 768 × 576, respectively.

For quantitative comparison, four metrics—number of correct matches (NCM), root mean square error (RMSE) [35], precision, and recall [12], are used for evaluation. These metrics are defined as follows:

(27) NCM=CorrectMatches

CorrectMatches indicates the number of correct matches in the final match result.

RMSE is the root mean square error between the correct match and the reference match obtained by the alignment method, the smaller the value of RMSE, the more accurate the alignment method is. RMSE can be expressed as follows:

(28) $$RMSE=\sqrt{\frac{1}{N}\sum_{I=1}^{N}{\left(x_{i}-x_{i}^{\prime }\right)}^{2}+{\left(y_{i}-y_{i}^{\prime }\right)}^{2}}$$

where $\left(x_{i},y_{i}\right)$ and $\left(x_{i}^{\prime },y_{i}^{\prime }\right)$ are the coordinates of the *i*th matched key point pair. *N* denotes the total number of match points. Precision is the ratio of the number of correct matches to the total number of matches, the larger the value, the higher the accuracy of the feature-matching method. Recall indicates the ability of the algorithm to extract correctly matched feature points from different source images, the larger the value, the more adaptive the alignment method is in distinguishing feature points.

(29) $$\begin{array}{cc} Precision & =\frac{CorrectMatches}{CorrectMatches+FalseMatches} \end{array}$$

(30) $$\begin{array}{cc} Recall & =\frac{CorrectMatches}{CorrespondingFeatures} \end{array}$$

where CorrespondingFeatures is the number of correct matches among the assumed feature points. When analysing the matching results, we use a manually computed transformation matrix to compute the projected residuals for each matching pair. If it is less than 6, the corresponding matching pair can be recognised as a correct match.

### 4.2. Comparison of Results with Other Methods

**Figure 7:**
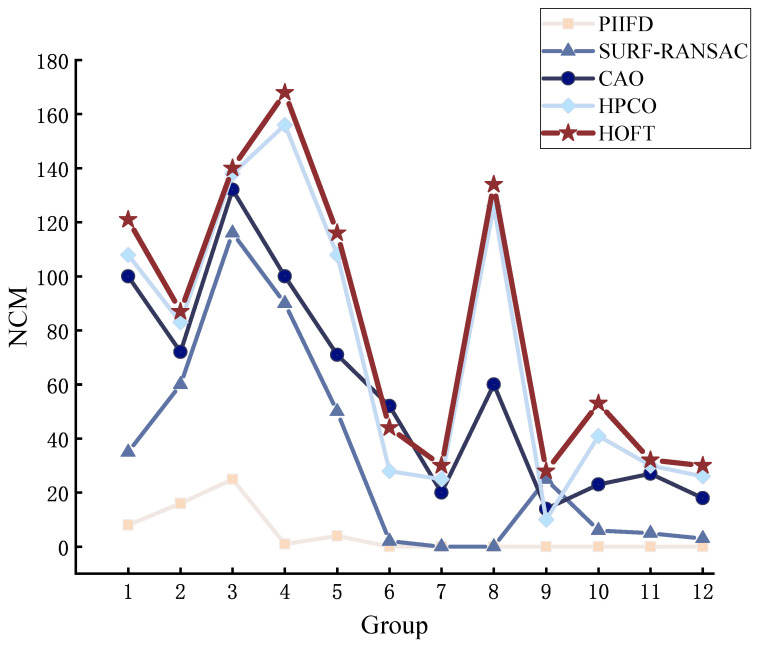
Evaluating the performance of methods for NCM.

We perform image alignment on visible and infrared image pairs in the dataset to evaluate the overall performance. All the image pairs in the dataset are aligned using PIIFD, SURF-RANSAC, CAO, HPCO, and our proposed HOFT method. From the data results, we can see that the HOFT method shows good performance in all the metrics. As shown in Figure 7, the NCM obtained under the HOFT method is larger, especially noticeable in groups 1–5. The PIIFD descriptor itself is not scale-variable, which makes it difficult to cope with significant scale and viewpoint variations, and it is not able to detect a sufficient number of highly repetitive feature points, resulting in an overall poorer matching result.

**Figure 8:**
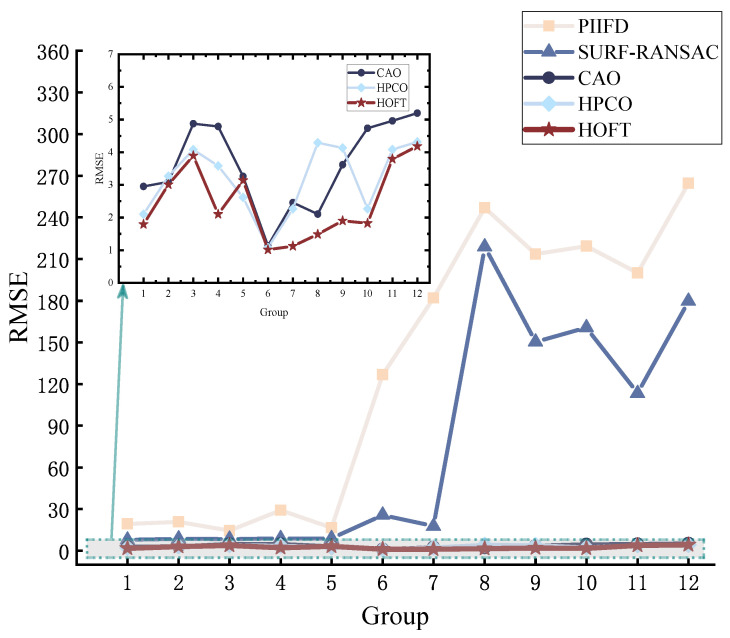
Evaluating the performance of methods for RMSE.

In terms of RMSE, as shown in Figure 8, the values of CAO, HPCO, and HOFT are overall significantly lower than those of PIIFD and SURF-RANSAC methods, especially in experiments 6–12, where the PIIFD and SURF-RANSAC methods are unable to accurately align images with obvious scale differences and viewpoint differences. On the contrary, the HOFT method has good accuracy regardless of the differences between image pairs.

**Figure 9:**
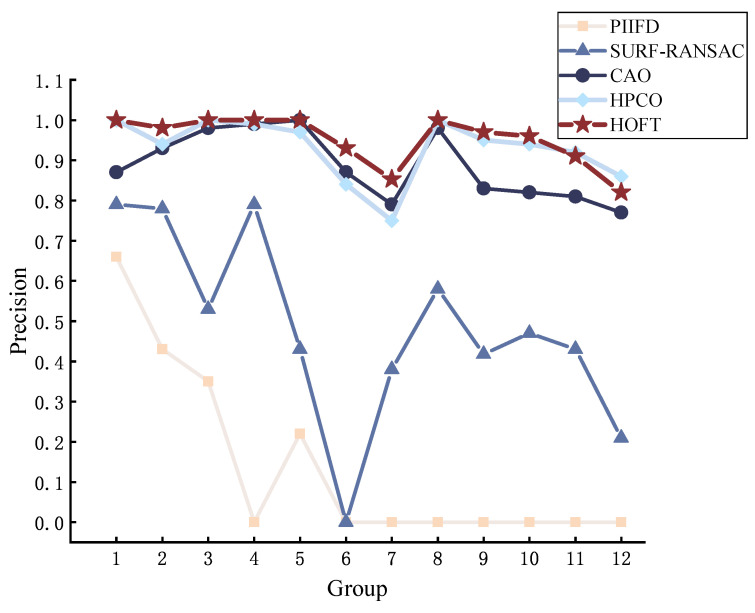
Evaluating the performance of methods for precision.

**Figure 10:**
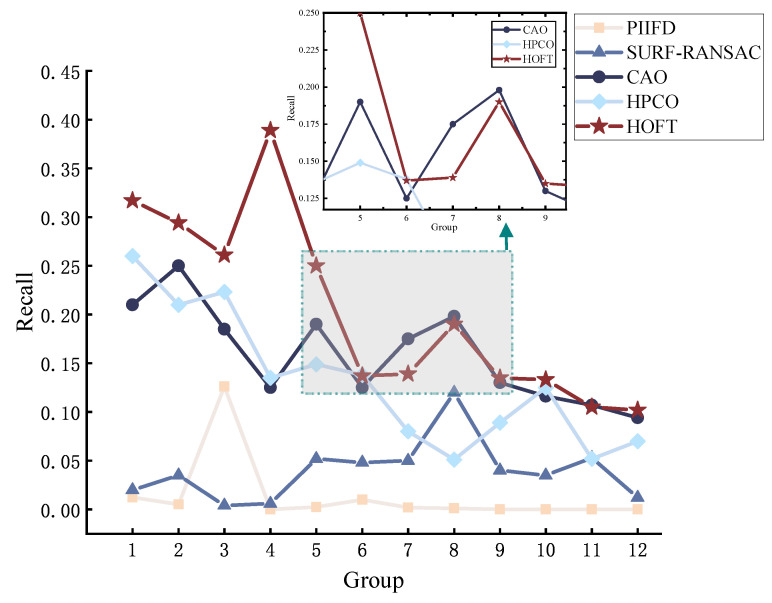
Evaluating the performance of methods for recall.

The performance of the comparatively evaluated methods in terms of precision and recall are shown in Figure 9 and Figure 10. By comparing precision and recall, it can be seen that the HOFT method still has stable matching performance, and the results show that the method optimises the positional error on the feature matching method to improve the matching accuracy, and the alignment method distinguishes the feature points with high adaptability, which can match each feature point better. However, in terms of evaluating the repeatability, image pairs 7–8 are slightly better than other methods under the CAO method, and the CAO method can obtain good matching performance by its principal direction orientation when the features are more obvious.

The average running time per image pair under PIIFD, SURF-RANSAC, CAO, HPCO, and HOFT methods are 23.68 s, 12.37 s, 7.53 s, 20.57 s, and 9.78 s. We can find that the average running time of the proposed HOFT method is less than most of the methods. In general, the HOFT method saves more than half of the time to compute the feature descriptors than the traditional methods. As shown in the Table 2, the time required for the run is given in the table. Although the time is slightly higher than the CAO method, it has been significantly improved over other methods.

**Figure 11:**
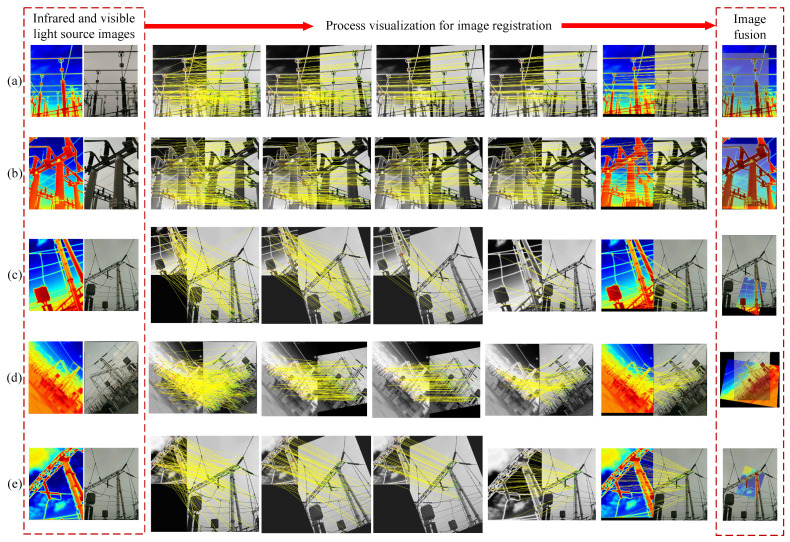
Visualisation of the visual matching effect of this method in different power scene information. (**a**) represents the visualisation effect of the alignment of power lines. (**b**) describes the impact of alignment visualisation of power equipment. (**c**) represents the alignment visualisation effect of image pairs with obvious scale differences. (**d**) represents the effect of alignment visualisation of image pairs with obvious rotation differences. (**e**) denotes the impact of alignment visualisation of image pairs with scale, rotation, and viewpoint differences. The visualisation effect of image fusion is given in the rightmost column to illustrate the accuracy and feasibility of our image registration method.

To visualise the matching results of our method, matching examples are given in Figure 11. As shown in the figure, we selected representative image pairs of each type to visualise the registration process. Figure 11a,b are image pairs with no obvious differences, Figure 11a mainly represents the information of power lines and Figure 11b mainly represents the information of power equipment. All of them can find more correct matching point pairs in the registration process, and it can be seen from the final fusion effect that the corresponding positions of the infrared and visible images overlap, which achieves the purpose of the application of image registration. Figure 11c–e are image pairs with significant differences; while Figure 11c mainly represents information with large scale differences, Figure 11d represent information with large rotation differences, and Figure 11e represents information with large differences in scale, rotation, and viewpoint.

From the visualization results, we can see that the overall number of matched pairs is less than that of the no-significant difference type. However, by combining the results of group 8 in Figure 7 with the analysis of the visualisation process in (d), excellent performance can be achieved and a large number of correct matching pairs can be found under rotational differences. The HOFT method still has good robustness and effectiveness under the cases of scale, rotation, and view angle differences, and the effect is more prominent, especially under the case of rotation. The results show that the intuitive evaluation of image fusion is effective, and the infrared and visible image registration can quickly and efficiently find the spatial mapping relationship between the two, align the same target in the image, and present the information that different images have in the same spatial coordinate system in the fused image.

### 4.3. Evaluation of Improved Feature Matching

**Figure 12:**
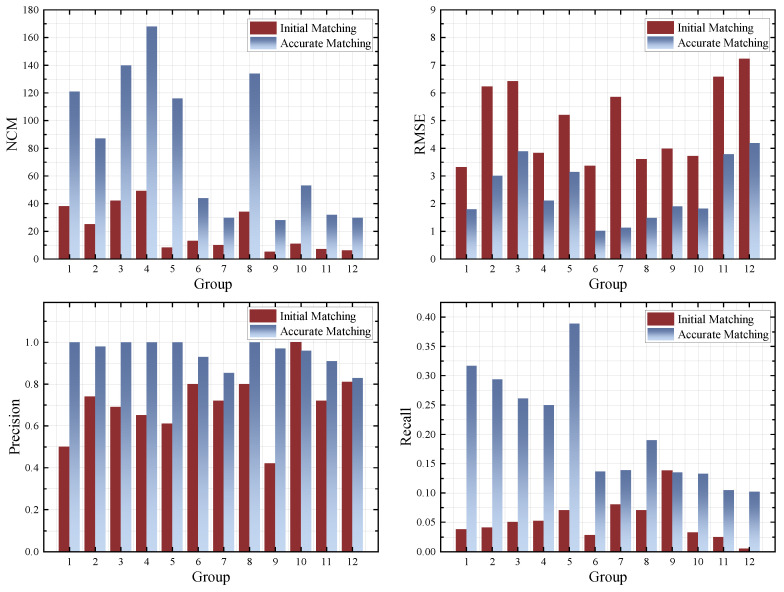
Quantitative comparison between initial and accurate matching.

In this paper, the intrinsic information position, scale, and orientation information of each key point is considered to increase the number of correct matches and to optimise the positional error of the final matched points. When the erroneous feature points are eliminated by Euclidean distance screening with error compensation, we obtain an initial match. Then, the process of refining the feature points is executed by adaptive thresholding with the RANSAC algorithm to achieve dense and accurate matching results. We use a more robust and detailed approach for dense feature point matching and overcome the problem of inaccurate matching due to inherent information location bias. For quantitative evaluation, we compare the matching effect before and after initial matching and accurate matching. As shown in Figure 12. In this paper, the proposed feature matching can greatly improve the performance of correct feature point matching.

## 5. Conclusions

To detect representative key points, firstly, the edge information of the image is extracted by the Sobel detection operator and scale invariant feature points are extracted using the CSS corner point detector. Secondly, the gradient information distribution property with a directional gradient histogram is introduced to establish the HOFT feature transformation descriptor. Finally, to improve the performance of the final correct matching, a strategy of intrinsic information error compensation is introduced for initial matching. Adaptive thresholding is incorporated into the RANSAC algorithm to realise exact matching to obtain more accurate results. Although our results show that the proposed method can robustly match feature points in infrared and visible images, it still suffers from the problem of low feature learning efficiency. Deep learning has powerful feature learning capabilities. Combining deep learning methods with feature-based approaches may lead to several potential research directions.

1. Deep optimisation of feature extraction and description: More fine-grained and robust extraction and description of features for infrared and visible images avoids the tedious process of manually selecting and designing features, and at the same time is able to better handle complex image transformations.
2. Deep learning-driven similarity measurement and matching strategy: Deep learning has obvious advantages such as being completely data-driven and being able to extract deep semantic features of images. Deep learning-based matching strategies are able to find the best correspondence between two images.
3. End-to-end alignment network design: The end-to-end alignment network can simplify the alignment process, improve the alignment efficiency, and potentially achieve higher alignment accuracy.
4. Hybrid model and multimodal fusion strategy: Combine traditional feature-based methods with deep learning algorithms to form a hybrid model. At the same time, multimodal fusion strategies for infrared and visible images are explored to make full use of information from both images.
5. Small-sample learning and migration learning: Explore deep learning training strategies based on small samples, such as migration learning and semi-supervised learning, in the case of a lack of datasets.

However, since IR spectra are usually low resolution and blurry, it is not easy to reliably extract detailed features such as key points, which are not easy to find in deep learning architectures. Future work will invoke deep learning methods in conjunction with the currently proposed method to enhance the accuracy of infrared and visible image alignment.

## Figures and Tables

**Table 1:** Details of the dataset.

Characteristic	Group	Image Pair Details and Differences
No significant difference in scale, rotation, and viewpoint	1	Image Information of Power Lines
	2	
	3	Image Information of Power Equipment
	4	
	5	
With significant differences in scale, rotation, and viewpoint	6	Differences in Scale are Evident
	7	
	8	Differences in Rotation are Evident
	9	
	10	Differences in Scale, Rotation, and viewpoint
	11	
	12	

**Table 2:** Running time per image pair using the HOFT method.

**Group**	1	2	3	4	5	6
**Running Time**	8.7681 s	7.8447 s	7.9695 s	8.5594 s	7.5707 s	7.9513 s
**Group**	7	8	9	10	11	12
**Running Time**	8.4584 s	8.1070 s	9.2244 s	14.9479 s	14.1430 s	13.7984 s

## Data Availability

The data and materials that support the findings of this study are available from the corresponding author upon reasonable request.

## Abbreviations

The following abbreviations are used in this manuscript:

CSS	Curvature Scale Space
HOG	Histogram of Orientation Gradients
SIFT	Scale-Invariant Feature Transform
FLANN	Fast Library for Approximate Nearest Neighbours
RANSAC	Random Sample Consensus
HOFT	Histogram of Orientation Gradients Combined with Feature Transform
MI	Mutual Information
NCC	Normalised Cross-Correlation
SURF	Speeded-Up Robust Features
PIIFD	Partial-Intensity Invariant Feature Descriptor
EAT	Enhanced Affine Transform
